# Etiologies of altered level of consciousness in the emergency room

**DOI:** 10.1038/s41598-022-09110-2

**Published:** 2022-03-23

**Authors:** Keun Tae Kim, Jae Cheon Jeon, Chang-Gyu Jung, Jung A. Park, Jong-Geun Seo, Doo Hyuk Kwon

**Affiliations:** 1 Department of Neurology, Keimyung University School of Medicine, Daegu, South Korea; 2 Department of Emergency Medicine, Keimyung University School of Medicine, Daegu, South Korea; 3 Department of Internal Medicine, Keimuyung University School of Medicine, Daegu, South Korea; 4 Department of Neurology, Daegu Catholic University School of Medicine, Daegu, South Korea; 5 Department of Neurology, School of Medicine, Kyungpook National University, Daegu, South Korea; 6 Department of Neurology, Yeungnam University College of Medicine, 170 Hyeonchungno, Nam-gu, Daegu, 42415 South Korea

**Keywords:** Neurology, Signs and symptoms, Neuroscience, Diseases of the nervous system, Diagnosis, Prognosis, Diseases, Cardiovascular diseases, Endocrine system and metabolic diseases, Neurological disorders

## Abstract

Altered levels of consciousness (ALCs) is a challenging issue; however, data describing its etiology and frequency are lacking. This study aimed to clarify and classify the etiologies of ALCs in the emergency room (ER) and to evaluate their destinations and the form of discharge. This retrospective study included patients with an ALC who visited the ER of a university hospital between January 2018 and December 2020. The cause and classification of the ALCs were carefully determined by a consortium of board-certified faculty members in emergency medicine, internal medicine, and neurology. The reference point for determining the etiology of ALC was discharge from the ER. In total, 2028 patients with ALCs were investigated. More than half (1037, 51.1%) visited the ER between 9:00 and 18:00. The most common etiology was systemic infection (581, 28.6%), followed by metabolic causes (455, 22.4%), and stroke (271, 13.4%). The two leading etiologies were extracranial and had a majority of the cases (1036, 51.5%). The overall mortality rate was 17.2%. This study provides fundamental information on ALC in the ER. Although intracranial etiologies have been foregrounded, this study demonstrated that extracranial etiologies are the main cause of ALC in the ER.

## Introduction

### Background

New-onset altered level of consciousness (ALC) refers to the change or deterioration of attention or arousal that is not caused by physiological drowsiness^1,2^. ALC is a frequently used phrase in the emergency rooms (ERs) that encompasses any change in the patient’s consciousness level from baseline. A proficient clinician is aware of the differential diagnosis of ALC in the ER; however, there is a lack of data describing its etiology and frequency in real-world medicine.

Although ALC has been a major factor in ERs, few studies have assessed the etiology of ALC in the ER. In early 2000, Kanich et al.^1^ evaluated 317 patients with ALC at a university hospital in the U.S. and reported that the common causes of ALC in the ER were neurological (28%), toxicologic (21%), and trauma-related (21%). Kekec et al.^3^ reviewed 790 patients with ALC in the ER at a university hospital in Turkey and reported a high prevalence of neurological problems (71.6%) leading to ALC, followed by trauma (10.4%), metabolic causes (6.1%), and cardiopulmonary disorders (6.2%). Xiao et al.^4^ evaluated 1,831 patients with ALC at a tertiary referral center in China and reported that the etiologies of ALC in the ER included pharmacologic and toxicologic causes (drug or alcohol) (23.0%), stroke (19.3%), systemic and organ dysfunction (14.5%), and infection (9.1%). Völk et al.^5^ reviewed 212 patients with ALC at a tertiary care hospital in Germany for 1 year and reported that the leading cause of ALC in the ER was stroke (24%), followed by systemic infections (12%), epileptic seizures (12%), and psychiatric diseases (8%). There has been a discrepancy in the classification of the etiology as well as the prevalence of ALC in the ER, which is due to the non-specificity of the ALC, the study population, and the definition of ALC.

### Goal of this investigation

ALC may be a symptom with a clear cause; however, it is often impossible to identify the cause, as it may be a temporary symptom of a dynamically changing disease. Since ALC in the ER remains challenging, we attempted to clarify the etiologies and their classification. This study aimed to clarify and classify the etiologies of ALC in the ER and evaluate their destinations and form of discharge.

## Methods

### Study design and selection of participants

This retrospective observational study included patients with ALC who visited the ER of a university hospital between January 2018 and December 2020. At this university hospital, the Glasgow Coma Scale (GCS) scorer was the first clinician to examine the patient and was the attending chief resident or board-certified faculty member in the emergency medicine department. All patients' GCS scores were recorded at the time of entering the ER. All patients other than those with scores of 15 (15 indicates that they were fully awake) were reviewed based on their medical records from the time they entered the ER to their discharge from the hospital. Patients under 19 years of age, those with cardiac arrest, and those who died on arrival were excluded. A revisit 24 h or more after the last discharge was regarded as a new visit. Regardless of the reason for admission, ALC cases that presented during hospitalization were excluded to investigate newly developed ALC cases only. This study was approved by the ethics committee of the Keimyung University Dongsan Medical Center (No. 2021-03-046), and the requirement for written informed consent was waived due to the retrospective study design. All methods were performed in accordance with the relevant guidelines and regulations.

### Methods of measurement

In each patient, the cause of ALC was determined and classified case by case through a discussion in a consortium consisting of board-certified faculty members in emergency medicine, internal medicine, and neurology with different affiliations. The consortium held monthly meetings to review the medical records in detail, as presented by all members. The patients’ medical records, including age, sex, ER visit time, vital signs, past and present history, treatment, tentative diagnosis, destination from ER, physical examination, neurological examination results, electrocardiogram, electroencephalography, laboratory results such as arterial blood gas analysis and cerebrospinal fluid (CSF) analysis, and imaging studies such as chest radiography, computed tomography (of the brain, neck, chest, abdomen, and pelvis), and brain magnetic resonance imaging data were reviewed. The reference point for determining the etiology of ALC was discharge from the ER.

### Data analysis

The statistics used were mostly descriptive. Student’s t-test was used to compare the prognosis of ALC in the ER. The statistical tests were two-tailed and considered statistically significant at *p* < 0.05. IBM SPSS ver. 22.0 (IBM Corp., Armonk, NY, USA) was used for analysis.

## Results

### Patient characteristics

**Figure 1:**
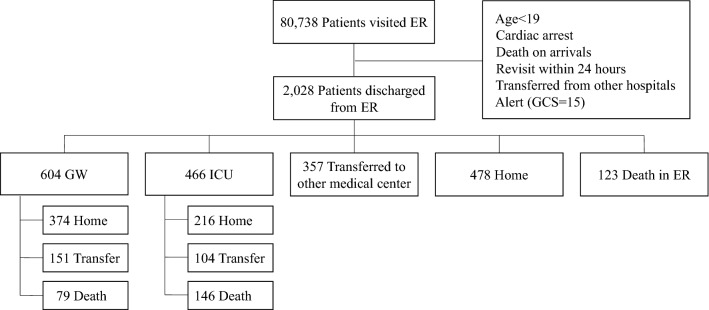
Enrollment, dispositions, and the form of discharge from the hospital. *ER* emergency room, *GCS* glasgow coma scale, *GW* general ward, *ICU* intensive care unit.

The total number of ER visits was 80,738. After screening for GCS scores of 15, we identified 2028 eligible patients with ALC in the ER. Their mean age was 68.33 ± 16.31 years (954 women, 47.0%) (Table 1, Fig. 1). A large proportion of the patients were in their 70 s (546, 26.9%), followed by their 80 s (506, 25.0%), and 60 s (358, 17.7%). The number of patients in their 70 s or older was 1,128, accounting for 55.6% of the total. The average length of stay time in the ER was 20 h and 11 min (20:11 ± 22:48, HH:MM). Stroke patients had the shortest stay time (6:33 ± 9:01), while the etiology of the longest stay time was systemic infection (25:34 ± 27:24), which included patients with the longest stay time (326:26).

**Table 1:** Demographic characteristics of the patients with altered level of consciousness in the emergency room.

	N = 2028 (100%)
Sex (Female) (%)	954 (47.0)
Age (years) (%)	
≤ 29	59 (2.9)
30–39	80 (3.9)
40–49	140 (6.9)
50–59	262 (12.9)
60–69	359 (17.7)
70–79	546 (26.9)
80–89	506 (25.0)
≥ 90	76 (3.7)
Length of stay in emergency room (HH:MM)	20:11 ± 22:48
Min ~ Max	00:04 ~ 326:26
Systemic infection	25:34 ± 27:24
Metabolic cause	21:05 ± 22:41
Stroke	6:33 ± 9:01
Toxicity	18:18 ± 20:49
Cardiogenic & vascular	14:19 ± 17:04
Seizure	17:28 ± 21:25
Psychiatric disorder	12:11 ± 13:33
TBI	17:47 ± 38:44
CNS infection	17:52 ± 15:35
Undetermined	10:19 ± 11:53

### Etiologies of ALC in ER

**Figure 2:**
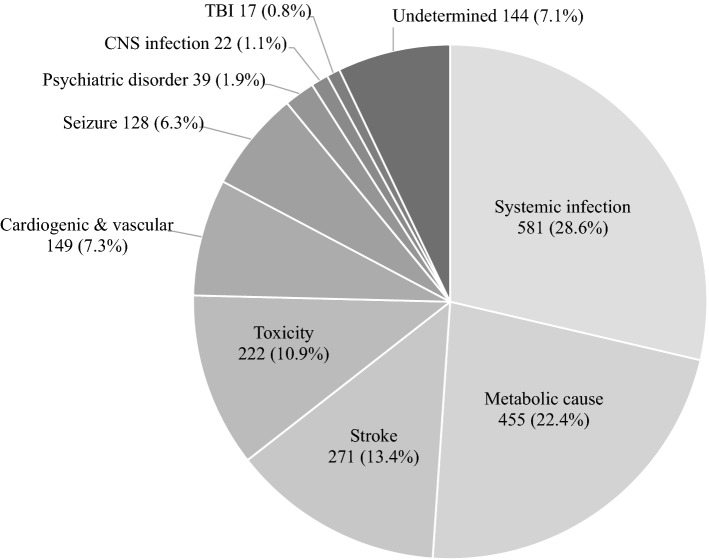
The etiologies of altered level of consciousness in the emergency room. *CNS* central nervous system, *TBI* traumatic brain injury.

The etiologies identified and classified by the authors through medical records in the ER are as follows: systemic infection, metabolic cause, stroke, cardiogenic & vascular cause, seizure, toxicity, psychiatric disorder, traumatic brain injury (TBI), central nervous system (CNS) infection, and undetermined (Table 2 and Fig. 2). Systemic infection was the most common etiology of ALC in the ER (581, 28.6%), which encompassed infectious conditions with the exclusion of CNS infection through CSF analysis (e.g., septic shock and sepsis), and 22 (1.1%) CNS infections were identified by CSF pleocytosis. In all patients with ALC in the ER, CSF analysis is a routine investigation performed to establish a treatment strategy or dose of medication; therefore, it should be performed before discharge from the ER to confirm the etiology of ALC. If CSF analysis was not performed in ALC patients with suspected systemic infection, the cause of their ALC in the ER would be classified as undetermined. Metabolic cause was the second most common etiology, presenting in 455 cases (22.4%). The clinical correlation between clinical features and laboratory test results is crucial to identify metabolic causes, such as hypoglycemia, diabetic ketoacidosis, hyponatremia, diabetic ketoacidosis, uremic encephalopathy, and hyperammonemia. The third etiology was stroke (271, 13.4%), and the symptoms and/or signs of cerebrovascular attack were ascertained by neuroimaging. This etiology includes cerebral infarction and intracranial hemorrhage, with the exception of TBI. There were 222 (10.9%) patients with ALC in the ER due to toxic agents such as pesticides, anaphylaxis, carbon monoxide, chemical agents, overdose of sedatives, and adverse effects of medications (e.g., antiepileptic drugs). In 149 (7.3%) cases, the ALC in the ER resulted from cardiogenic & vascular causes, such as myocardial infarction, ventricular tachycardia, aortic dissection, impending rupture of aortic aneurysm, and cardiac tamponade. With or without a history of epilepsy, 128 patients (6.3%) had ALC due to seizures, including epileptic as well as psychogenic non-epileptic seizures (PNES). The number of ALC patients that were in the ER due to psychiatric disorders and TBI was 39 (1.9%) and 17 (0.8%), respectively. Psychiatric disorder refers to any psychiatric disorder that causes ALC, except PNES. This etiology was identified through face-to-face interviews with an attending psychiatrist in the ER after reasonable exclusion of alternative causes. ALC that is associated with trauma, which is identified based on the patient’s present history and neuroimaging, is classified as TBI and includes concussions. The cause of ALC remained undetermined in 144 (7.1%) patients despite intensive evaluations being performed for 1 or 2 days in the ER. This etiology includes not only cases with insufficient evaluation but also cases with two or more (i.e., concurrent) etiologies.

**Table 2:** The etiologies of altered level of consciousness in the emergency room (N = 2028).

Etiology	n (%)
Systemic infection	581 (28.6)
Metabolic cause	455 (22.4)
Stroke	271 (13.4)
Toxicity	222 (10.9)
Cardiogenic & vascular	149 (7.3)
Seizure	128 (6.3)
Psychiatric disorder	39 (1.9)
TBI	17 (0.8)
CNS infection	22 (1.1)
Undetermined	144 (7.1)

*TBI* traumatic brain injury, *CNS* central nervous system.

### Etiologies of ALC in ER by time zone

**Figure 3:**
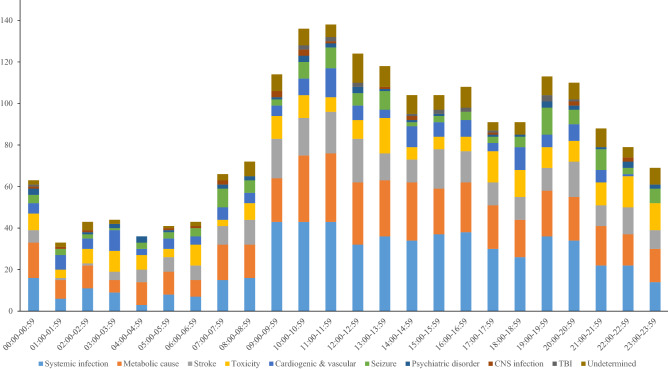
The etiology analysis by time zone. *CNS* central nervous system, *TBI* traumatic brain injury.

Table 3 shows the visit times of patients with ALC, as illustrated in Fig. 3. The greatest number of ALC patients [138 (6.8%)] visited the ER between 11:00 and 11:59. Between 9:00 and 17:59, 1,037 (51.1%) ALC patients visited the ER, with 512 (25.2%) visiting it during the 4-h period from 9:00 to 12:59. Throughout the period from 9:00 to 22:59, the leading cause of ALC in the ER was systemic infection. One hundred and twenty-nine patients, accounting for 22.2% of all systemic infections, visited the ER between 9:00 and 12:00. From 12:00 to 12:59, 21:00 to 21:59, and 23:00 to 23:59, undetermined cases accounted for more than 10% of the ALC patients in each period (11.3%, 10.2%, and 11.6%, respectively).

**Table 3:** The etiologies of altered level of consciousness in the emergency room by time zone.

	Total (N = 2028)	Systemic infection	Metabolic cause	Stroke	Toxicity	Cardiogenic & vascular	Seizure	Psychiatric disorder	CNS infection	TBI	Undetermined
00:00–00:59	63 (3.1)	16 (25.4)	17 (27.0)	6 (9.5)	8 (12.7)	5 (7.9)	4 (6.3)	3 (4.8)	1 (1.6)	1 (1.6)	2 (3.2)
01:00–01:59	33 (1.6)	6 (18.2)	9 (27.3)	1 (3.0)	4 (12.1)	7 (21.2)	3 (9.1)	0	1 (3.0)	0	2 (6.1)
02:00–02:59	43 (2.1)	11 (25.6)	11 (25.6)	1 (2.3)	7 (16.3)	5 (11.6)	2 (4.7)	1 (2.3)	1 (2.3)	0	4 (9.3)
03:00–03:59	44 (2.2)	9 (20.5)	6 (13.6)	4 (9.1)	10 (22.7)	10 (22.7)	1 (2.3)	2 (4.5)	0	0	2 (4.5)
04:00–04:59	36 (1.8)	3 (8.3)	11 (30.6)	6 (16.7)	7 (19.4)	3 (8.3)	3 (8.3)	3 (8.3)	0	0	0
05:00–05:59	41 (2.0)	8 (19.5)	11 (26.8)	7 (17.1)	4 (9.8)	5 (12.2)	3 (7.3)	1 (2.4)	1 (2.4)	0	1 (2.4)
06:00–06:59	43 (2.1)	7 (16.3)	8 (18.6)	7 (16.3)	10 (23.3)	4 (9.3)	4 (9.3)	0	1 (2.3)	0	2 (4.7)
07:00–07:59	66 (3.3)	15 (22.7)	17 (25.8)	9 (13.6)	3 (4.5)	6 (9.1)	9 (13.6)	2 (3.0)	2 (3.0)	0	3 (4.5)
08:00–08:59	72 (3.6)	16 (22.2)	16 (22.2)	12 (16.7)	8 (11.1)	5 (6.9)	6 (8.3)	2 (2.8)	0	0	7 (9.7)
09:00–09:59	114 (5.6)	43 (37.7)	21 (18.4)	19 (16.7)	11 (9.6)	5 (4.4)	3 (2.6)	1 (0.9)	3 (2.6)	0	8 (7.0)
10:00–10:59	136 (6.7)	43 (31.6)	32 (23.5)	18 (13.2)	11 (8.1)	8 (5.9)	8 (5.9)	3 (2.2)	3 (2.2)	2 (1.5)	8 (5.9)
11:00–11:59	138 (6.8)	43 (31.2)	33 (23.9)	20 (14.5)	7 (5.1)	14 (10.1)	10 (7.2)	2 (1.4)	1 (0.7)	2 (1.4)	6 (4.3)
12:00–12:59	124 (6.1)	32 (25.8)	30 (24.2)	21 (16.9)	9 (7.3)	7 (5.6)	6 (4.8)	3 (2.4)	0	2 (1.6)	14 (11.3)
13:00–13:59	118 (5.8)	36 (30.5)	27 (22.9)	13 (11.0)	17 (14.4)	4 (3.4)	9 (7.6)	1 (0.8)	1 (0.8)	0	10 (8.5)
14:00–14:59	104 (5.1)	34 (32.7)	28 (26.9)	11 (10.6)	6 (5.8)	10 (9.6)	2 (1.9)	1 (1.0)	2 (1.9)	1 (1.0)	9 (8.7)
15:00–15:59	104 (5.1)	37 (35.6)	22 (21.2)	19 (18.3)	6 (5.8)	7 (6.7)	3 (2.9)	1 (1.0)	0	2 (1.9)	7 (6.7)
16:00–16:59	108 (5.3)	38 (35.2)	24 (22.2)	15 (13.9)	7 (6.5)	8 (7.4)	4 (3.7)	0	0	2 (1.9)	10 (9.3)
17:00–17:59	91 (4.5)	30 (33.0)	21 (23.1)	11 (12.1)	15 (16.5)	4 (4.4)	3 (3.3)	1 (1.1)	1 (1.1)	1 (1.1)	4 (4.4)
18:00–18:59	91 (4.5)	26 (28.6)	18 (19.8)	11 (12.1)	13 (14.3)	11 (12.1)	5 (5.5)	1 (1.1)	0	0	6 (6.6)
19:00–19:59	113 (5.6)	36 (31.9)	22 (19.5)	11 (9.7)	10 (8.8)	6 (5.3)	13 (11.5)	3 (2.7)	0	3 (2.7)	9 (8.0)
20:00–20:59	110 (5.4)	34 (30.9)	21 (19.1)	17 (15.5)	10 (9.1)	8 (7.3)	7 (6.4)	2 (1.8)	2 (1.8)	1 (0.9)	8 (7.3)
21:00–21:59	88 (4.3)	22 (25.0)	19 (21.6)	10 (11.4)	11 (12.5)	6 (6.8)	10 (11.4)	1 (1.1)	0	0	9 (10.2)
22:00–22:59	79 (3.9)	22 (27.8)	15 (19.0)	13 (16.5)	15 (19.0)	1 (1.3)	3 (3.8)	3 (3.8)	2 (2.5)	0	5 (6.3)
23:00–23:59	69 (3.4)	14 (20.3)	16 (23.2)	9 (13.0)	13 (18.8)	0	7 (10.1)	2 (2.9)	0	0	8 (11.6)

*TBI* traumatic brain injury, *CNS* central nervous system.

### Dispositions of the patients with ALC from ER

The numbers of admissions to the general ward (GW) and intensive care unit (ICU) were 604 (29.8%) and 466 (23.0%), respectively (Table 4). Three hundred fifty-seven (17.6%) were transferred from the ER to another hospital. A hundred and twenty-three (6.1%) patients with ALC died in the ER without being admitted to the GW or ICU, or were transferred to another hospital (Table 4, Fig. 1). Although there might be multiple causes of death, the causes of the deceases-in-ER could be complications of ALC rather than comorbidities. For example, systemic infection resulting in shock, metabolic causes followed by multiorgan failure, cardiac arrests subsequent to arrhythmia, and sudden death due to rupture of the aortic aneurysm are all possible comorbidities. The most common etiology of decease-in-ER was systemic infection (n = 62, 50.4%), followed by metabolic causes (n = 23, 18.7%). In the 18 (14.6%) decease-in-ER cases, unstable vital signs precluded further evaluations and left the etiology undetermined (Table 4). While 478 (23.6%) patients who had recovered consciousness after entering the ER were sent home, 357 (17.6%) were transferred to another hospital due to the need for emergency surgery, the lack of a hospital ward, or the caregiver’s demand. Of the 1,070 admissions, 604 (29.8%) were to GWs and 466 (23.0%) were to the ICU.

**Table 4:** Dispositions of altered level of consciousness in the emergency room.

Dispositions	No (%) N = 2028	Systemic infection	Metabolic cause	Stroke	Cardiogenic & vascular	Seizure	Toxicity	Psychiatric disorder	CNS infection	TBI	Undetermined
GW	604 (29.8)	212 (35.1)	175 (29.0)	73 (12.1)	26 (4.3)	51 (8.4)	29 (4.8)	10 (1.7)	3 (0.5)	10 (1.7)	15 (2.5)
ICU	466 (23.0)	135 (29.0)	66 (14.5)	136 (29.2)	86 (18.5)	16 (3.4)	3 (0.6)	0	6 (1.3)	5 (1.1)	13 (2.8)
Transfer	357 (17.6)	141 (39.5)	80 (22.4)	47 (13.2)	14 (3.9)	4 (1.1)	35 (9.8)	2 (0.6)	9 (2.5)	2 (0.6)	23 (6.4)
Home	478 (23.6)	31 (6.5)	111 (23.2)	12 (2.5)	11 (2.3)	56 (11.7)	153 (32.0)	26 (5.4)	3 (0.6)	0	75 (15.7)
Death	123 (6.1)	62 (50.4)	23 (18.7)	3 (2.4)	12 (9.8)	1 (0.8)	2 (1.6)	1 (0.8)	1 (0.8)	0	18 (14.6)

*TBI* traumatic brain injury, *CNS* central nervous system, *GW* general ward, *ICU* intensive care unit.

Figure 1 shows the journey of patients from the ER to discharge from the hospital. Of the 604 patients admitted to the general ward, 374 (61.9%) were sent home, 151 (25.0%) were transferred to another hospital after acute management, and 79 (13.1%) died. Of the 466 patients admitted to the ICU, 216 (46.4%) were sent home, 104 (22.3%) were transferred to another hospital after acute management, and 146 (31.3%) died. A total of 1070 patients with ALC in ER were hospitalized, of which 225 (GW 79 + ICU 146, 21.0%) died. Eventually, 348 of 2028 (17.2%) patients with ALC died, including 123 deaths in the ER. Although the etiology classification was not a confirmative diagnosis and may not be the cause of death, mortality was higher in patients sent to the ICU than in those sent to the GW (29.2% vs. 15.6%, *p* = 0.000).

## Discussions

In real-world practice, ALC is an intricate issue in the ER, as it is a potentially life-threatening condition. Extensive history taking, ready-made medical pathways, due diligence by medical staff, prompt assessment, and appropriate approaches are needed. Not only may the level of consciousness change dynamically and continuously, but the identification of etiology is also often obscure. In addition, there are several synonyms for ALC, such as loss of consciousness, altered mental status, impaired consciousness, and altered level of consciousness. The variety of names suggests that ALC is a nonspecific sign or symptom with diverse etiologies.

The etiology of ALC in the ER has varied in previous studies. Although there have been studies on ALC in the ER, the populations included in the study were inconsistent. Previous studies have differentiated ALC based on chief complaints rather than objective findings on examinations^5–7^. In a previous study, the study duration was only 4 months^8^, and in another study of 14 days, only patients who visited during the daytime were considered^9^. Some studies only considered elderly patients aged 65 or 70 years or older^7,9^ or only included trauma patients^10^. In a previous study, a medical doctor did not evaluate the degree of ALC of the patients^5^. In some studies, the clinicians who participated in the evaluation were from a single department, such as the neurology or emergency medicine departments^1,6,8,10^. The etiology classification system of ALC in the ER is also inconsistent. In a previous study, the classification included neurological, trauma, endocrine/metabolic, cardio/pulmonary, infection, gynecological, and toxic causes^3^. In another study, the etiology of ALC in the ER included stroke, malignancy, and metabolic causes. Subsequently, metabolic causes were divided into hepatic encephalopathy, alcohol-related disorders, uremic encephalopathy, hypoglycemia, and electrolyte imbalance^6^. In addition, a previous study included transient general amnesia (TGA) in its classification^5^; however, it is well known that the level of consciousness is normal in TGA.

The aforementioned inconsistencies in study design and etiology classifications make it difficult to represent patients with ALC in the ER. To overcome these difficulties, we reviewed more than 2000 cases in the past 2 years and accepted conservative exclusion criteria. We reviewed all patients whose GCS score was not 15 at the moment they entered the ER, and consequently, all alert patients were excluded. Since it is often impossible to identify the cause, course, and baseline consciousness statuses of patients with preexisting ALCs, including major stroke, hypoxic brain damage, TBI, infection, depression, schizophrenia, and dementia, any ALCs that occurred during hospitalization at other hospitals were excluded. In addition, cases of cardiac arrest and DOA were excluded, as their causes remained unsolved. The consortium of board-certified faculty members in emergency medicine, internal medicine, and neurology conducted a detailed review of the medical records, and sufficient discussion was carried out to classify the etiologies of ALC in the ER. Consequently, this study identified 10 etiologies of ALC in the ER.

In previous studies^1,3,5,7–10^, intracranial etiologies (e.g., stroke or seizure) were the most common etiology of ALC in the ER, accounting for approximately one-third of cases. In this study, systemic infection was the leading cause, accounting for more than a quarter of cases, followed by metabolic causes, accounting for approximately one-fifth of cases. These two etiologies combined accounted for more than half of the ALC cases in the ER (n = 1036, 51.1%). In contrast, intracranial diseases accounted for 21.6% of the cases, including stroke, seizure, TBI, and CNS infections. Even if all intracranial cases were combined, the number was lower than that for metabolic causes (438 intracranial etiologies vs. 455 metabolic causes). However, that is not to say that intracranial etiologies do not matter. As the slogan "Time is brain" states^11,12^, prompt evaluations and immediate responses are paramount for intracranial etiologies such as acute ischemic stroke and status epilepticus. The short stay of stroke patients implies the importance and urgency of stroke in the ER. In this study, the average stay time of stroke was only 6.5 h, which is less than a quarter of that of systemic infection. Moreover, in real-world practice, the clinician in the ER can check vital signs and perform history taking as well as neurologic examinations prior to the results of laboratory or radiologic evaluations. Careful consideration and clinical judgment for intracranial etiology such as stroke are crucial in ALC in the ER, although intracranial diseases such as stroke and seizure rank next to systemic infection or metabolic causes.

The etiology of ALC in the ER remained undetermined in 7%, despite intensive evaluations being performed in the ER for a day or two. The diagnoses in the ER are sometimes provisional, not confirmative, and two or more causes cannot be identified and are often determined after inpatient treatment. For example, a patient in this study diagnosed with stroke in the ED was found to have multiple sclerosis after sufficient examination. In another example, the authors identified an anorexia patient with ALC who had hyponatremia of 118 mEq/L and hypoglycemia of 30 mg/dL. The cause of this patient’s ALC could not be determined; however, there is no doubt that this was classified as a metabolic cause. In another case, a patient with valproate for epilepsy visited the ER with a stuporous mentality; liver cirrhosis was first noticed, and hyperammonemia was revealed by abdominal computed tomography and blood tests, respectively. The cause of the ALC in this patient could not be specified and was classified as undetermined. In the present study, we identified a drowsy patient with CSF pleocytosis and CO_2_ retention. The tentative diagnosis was encephalitis; however, this patient was found to have neuromyelitis optica as an anti-aquaporin 4 antibody was detected. Indeed, clinicians in the ER should not assume confirmative diagnoses; instead, they should take the appropriate and necessary actions immediately. As in these examples, the presence of more than 7% of patients with unknown ALC etiologies suggests that emergency room care alone is insufficient for accurate diagnosis and treatment; additional medical approaches, including hospitalization, are required.

The distribution of the etiology of ALC may change depending on the time zone. This study demonstrated that the majority of patients with ALC visited the ER between 9:00 and 18:00, and a quarter visited in the forenoon. Of course, this study alone cannot determine the cause of the high incidence of ALC observed during the day; nonetheless, we can surmise that ALC patients cannot visit hospitals by themselves and can only visit hospitals with their family members’ help. There are standout hours in which patients with hypoglycemia are frequently seen in the ER and which coincide with time zones; additionally, the leading etiology of ALC in these cases is metabolic. Notably, the undetermined etiology was steady between 4 and 11%, except for a few hours during the night. This implies that, as mentioned above, undetermined is a significant etiology to be considered in almost all time zones.

The overall mortality rate of ALC cases in the ER was 17.2%. Since dispositions from the ER are determined according to the patient's condition and the clinician's judgment, the higher mortality of patients admitted to the ICU is not new. We suppose that disease severity, rather than etiology, would have affected mortality^13,14^. Since this study aimed to clarify and classify the etiologies of ALC in the ER and to evaluate their destinations and the form of discharge, the cause of mortality was beyond its scope. Future cohort studies are needed to provide additional clinical information. In this study, we considered 123 deaths in the ER. This may have been an unavoidable situation; however, there may be a problem in the patient flow system of this hospital^15^. Shortages in hospital beds should be addressed, as bed turnover rates in ERs, GWs, and ICUs greatly influence disposition from the ER^15–17^. The real issue is that the number of ALC patients among ER patients who needed inpatient treatment accounted for three-quarters (76.4%) of the total, consisting of 1070 patients admitted to this hospital, 357 who were transferred to another hospital, and 123 who died in the ER. In other words, approximately a quarter of the ALC cases in the ER were transient and allowed to be discharged home.

This study has several limitations. First, selection bias cannot be excluded because of the single-center retrospective study design; however, the comprehensive study inclusion criteria and large number of patients (> 2000) may redeem this limitation. Given that ALC in the ER per se is a critical situation that is regarded as a matter of urgency, a prospective study design has potential limitations. Second, since the reference point used to classify the etiology of ALC in the ER was discharge from the ER, the etiology classification was not conclusive. Third, this study did not provide details on the prognosis of ALC in the ER. Despite these limitations, it is noteworthy that the etiologies of the ALC cases in the ER were investigated and determined through careful discussion in the consortium, and fundamental information about ALC was provided in the ER. Furthermore, although previous studies have focused on the intracranial etiology of ALC in the ER, this study demonstrated that extracranial ALC is the predominant cause. Further studies are needed to confirm the utility and relevance of the etiological classification suggested in this study and to evaluate the prognosis of ALC in the ER.

ALC cases in the ER present with protean manifestations. It can have diverse etiologies, and not all causes can be identified in the ER. In the real world, there can be two or more causes concomitantly, and sometimes even initiation of the investigation is impossible. These are obstacles to the study of ALC in ER research, which is why further studies are required.

## Data Availability

The datasets used and/or analyzed during the current study are available from the corresponding author on reasonable request.
